# Violence in emergency services and preventative measures: results from an online survey from Germany

**DOI:** 10.1007/s11739-025-03994-4

**Published:** 2025-06-06

**Authors:** Ana Zhelyazkova, Matthias Bonigut, Eva Jansen

**Affiliations:** 1 Institut für Notfallmedizin und Medizinmanagement (INM), LMU Klinikum, LMU München, Munich, Germany; 2 Institut für Sozialmedizin, Epidemiologie und Gesundheitsökonomie der Charité, Universitätsmedizin Berlin, Berlin, Germany

**Keywords:** Emergency services, Violence, Healthcare workers, Violence prevention

## Abstract

Violence in emergency medical services (EMS) presents a critical issue, yet data on its extent and on effective preventative measures are lacking. We aim to explore violence experiences and opportunities for prevention from the actor-centered perspective of EMS workers in Bavaria, Germany. We conducted a questionnaire-based online survey of EMS workers (September 2023–January 2024), comprising standardized items and open questions on experiences and perceptions of workplace violence. In this context, we collected data on requested preventive measures. Mixed-methods analysis was applied, including descriptive statistics and qualitative thematic analysis. Of 224 completed questionnaires, 38.4% reported to have experienced physical and 57.6% non-physical violence at work during the past 12 months. Experiences of physical violence were associated with the female gender, while non-physical violence showed an inverse association with age and seniority. Participants also reported physical (0.022%) and non-physical violence (17.9%) from colleagues. Only a third noted that there are established reporting procedures for violent incidents in their workplace, thereof over a third had refrained from submitting a report despite experiencing violence. Among the most effective and urgently requested preventive measures, respondents listed communication-based de-escalation and crisis management as well as improvement of working conditions including managers’ leadership competencies. Results’ interpretation is impeded by the limited response. Physical and non-physical violence are prevalent in the workplace context of EMS in Germany. An improvement in the working conditions, which should include transparent and effective reporting mechanisms as well as communication-based prevention strategies is explicitly requested by employees.

**Supplementary Information:**

The online version contains supplementary material available at 10.1007/s11739-025-03994-4.

## Introduction

Workplace violence presents a major issue in healthcare services worldwide [1]. According to a previously published meta-analysis, approximately 62% of the health workforce experience violence at work [2]. Of those, more than 42% have experienced non-physical and over 24% physical violence. Experiences of violence within the workplace setting contribute to dissatisfaction and intention to leave, thus ultimately increasing the shortage in skilled workers [1, 3–5].

Among healthcare workers, those with considerable patient contact, such as in emergency care, are particularly exposed to malignant behavior from patients, bystanders, colleagues and managers [3, 4, 6–8]. Although the overall quantity and quality of evidence remain limited, the lack of data on violence experienced by emergency medical services (EMS) workers is particularly challenging due to recall and interpretation bias as well as low response rates [6, 9, 10]. Additionally, previous analyses have observed a compelling rate of underreporting workplace violence among the emergency services workforce, further impeding a structured overview of the problem and, consequently, developing, implementing and evaluating prevention strategies [4, 11].

Our work, therefore, aimed to explore German EMS workers’ experiences of violence in the workplace in terms of prevalence, situational characteristics and associated factors, reporting behavior as well as consequences. We explored the reasons for workplace violence as perceived by the EMS personnel as well as their preferences for preventive measures. For the purposes of this work, we considered EMS workers as personnel trained and actively employed in emergency services, such as trainees, qualified drivers, emergency medical technicians, paramedics and emergency physicians.

## Methods

Within the frames of this project, we adopted the definition of workplace violence by the World Health Organization (WHO): “Incidents where staff are abused, threatened or assaulted in circumstances related to their work, including commuting to and from work, involving an explicit or implicit challenge to their safety, well-being or health” [1].

We designed a voluntary, anonymous and online-based structured questionnaire informed by the validated questionnaire on workplace violence in the health sector by the Joint Programme on Workplace Violence in the Health Sector [12]. The questionnaire was supplemented with questions collecting information relevant to the German emergency medicine landscape and allowing for comparisons to previously published studies on the topic from Germany. The title of the survey as well as the invitation to participate were framed neutrally: “Conditions and barriers of working in emergency rescue services”. The questionnaire was divided into five sections: Employment situation (1), Questions on physical and sexual violence in the workplace (2), Questions on non-physical violence, including gender-based, sexualized, racist, or religious harassment and insults (3), Questions on prevention measures (4), and Sociodemographic data (5). The survey was aimed at EMS workers in Germany, however, we note that the number and characteristics (gender, age, allocation, qualifications) of the target population are unknown.

The primary outcome of the project was defined as a qualitative and quantitative assessment of the participants’ perception of violence in the work setting, where the source of violent behavior was not limited and can refer to the whole social context of professional activity. Violence can therefore include the interaction with a patient, patient’s family or friends, uninvolved bystanders as well as a colleague or manager. The questionnaire provided definitions for physical and non-physical violence translated into German from the questionnaire on workplace violence in the health sector by the Joint Programme on Workplace Violence in the Health Sector [12]. We disseminated the survey using two main strategies: invitation to participate sent by e-mail through the management of the emergency services organizations (such as Malteser, Johanniter, and Red Cross) (1), Facebook groups of the EMS community and LinkedIn (2). The survey was available to participants between September 7, 2023 and January 18, 2024.

The questionnaire was implemented with LimeSurvey Version 5.5.2 + 230,109. The analyses were executed using IBM SPSS Statistics Version: 29.0.0.0 (241). Quantitative data was analyzed using descriptive statistics, associations were tested using the Pearson Chi2 Test or Spearman’s rank correlation depending on the variables’ coding. We explored factors associated with having experienced physical or non-physical violence with binomial logistic regression and the associated factors with perceived trends in violence using ordinal logistic regression. Questions in open-text format were categorized into themes using the inductive content analysis described by Elo and Kyngäs [13]. Initial raw data review and category construction were conducted by one coder, whereas data classification and coding were conducted by two independent coders. Discrepancies in coding as well as disagreements regarding the categories’ description and structure were discussed and resolved between the two coders.

## Results

Of 397 submitted forms, 224 were fully filled out. The analyses conducted for this publication were based solely on the fully completed questionnaire forms (Table 1).

**Table 1:** Sociodemographic characteristics of the participants with fully completed questionnaire forms

VARIABLE	*n*	%	In your opinion, has the frequency of violence in the emergency services increased or decreased in recent years?°
Age			0.218
Younger than 24 years	51	22.8	
25–29 years	56	25.0	
30–34 years	35	15.6	
35–39 years	21	9.4	
40–44 years	20	8.9	
45–49 years	23	10.3	
50 years or older	18	8.0	
Gender			0.018*
Male	165	73.7	
Female	59	26.3	
Immigration background			0.422*
No	212	94.6	
Yes—born in the EU	5	2.2	
Yes—born in Germany with family migration background	7	3.1	
Yes—born outside the EU	0		
Province°			0.310*
Bayern	190	84.8	
Other	34*	16.2	
Type of emergency service area			0.036
Predominantly rural	33	14.7	
Predominantly urban	47	21.0	
Rural and urban (mixed)	66	29.5	
Exclusively urban (incl. metropolitan)	78	34.8	
Qualification (binary)**			0.405*
Non-paramedic	98	43.8	
Paramedic	126	56.3	
Years of experience in emergency medicine			0.312
Less than 1 year	6	2.7	
2–4 years	52	23.2	
5–9 years	68	30.4	
10–15 years	33	14.7	
More than 16 years	65	29.0	
Years of working for current employer			0.452
Less than 1 year	27	12.1	
2–4 years	65	29.0	
5–9 years	61	27.2	
10–15 years	30	13.4	
More than 16 years	41	18.3	
Mode of employment			0.316
Full-time	148	66.1	
Part-time°°	68	30.4	
Voluntary work	8	3.6	
°°Part-time working hours in percentage (*n* = 68)			0.199
< = 20%	20	8.9	
21–49%	30	13.4	
50–79%	16	7.1	
> = 80%	2	0.9	
Family constellation (binary)			0.234
Children	68	30.4	
No children	156	69.6	
Family constellation (ordinal)			0.426
No children	156	69.6	
1 child	21	9.4	
2 children	34	15.2	
3 or more children	13	5.8	
Of those: participants with at least one child…			0.059
Under the age of 12	52	23.2	
Above the age of 12***	16	7.1	

*The following analyses were conducted using a Pearson Chi2 test due to the nominal coding of the respective variable; for all other associations, Spearman was used

**Qualifications of the cohort: Trainee = 19, Qualified driver = 2, Emergency Medical Technician or similar = 75, Paramedic = 126. Emergency physician = 2

***Detailed distribution regarding education/care type: in kindergarten = 31, in school = 30, in other form of care = 2

°Baden-Württemberg = 8, Berlin = 2, Brandenburg = 1, Hessen = 3, Mecklenburg-Vorpommern = 1, Niedersachsen = 7, Nordrhein-Westfalen = 8, Rheinland-Pfalz = 2, Schleswig–Holstein = 2.

°°Correlation analyses were executed with the variable version in which the first two items are combined due to their lower frequency individually.

The variables for gender and type of emergency service area showed significant associations with the perceived tendency of violence. These were henceforth considered confounders in the following statistical analyses.

### Experience and perception of violence in emergency services as a work setting

We asked participants who had experienced physical and non-physical violence in the past 12 months to report the estimated number of violent acts they have experienced as well as to provide an estimate of the frequency of the type of situation in which these acts occurred. These are presented in Supplementary Tables 1 and 2.

Overall, 86 participants reported to have been exposed to physical violence in the past 12 months (Table 2; Median = 2.00), whereas 129 participants reported to have experienced non-physical violence (Median = 5.00).

**Table 2:** Frequency of violence experienced by EMS workers in the past 12 months

Violence experience in the past 12 months	Frequency
Physical violence	
Yes	86
No	138
Number of experiences of physical violence in the past 12 months	*x̅* = 3.62 *x̃* = 2.00 Range = 1–20
Non-physical violence	
Yes	129
No	95
Number of experiences of non-physical violence in the past 12 months	*x̅* = 22.98 *x̃* = 5.00 Range = 1–1290*

*Despite the upper range value having a potential for an outlier, we have decided to keep it to prevent a further reduction of the data sample. Furthermore, we cannot be certain of the deliberateness of the value as an answer as previous retrospective data collections have reported up to approx. 10.000 cases of verbal violence in a sample of 425 emergency services workers in Germany, where 3 participants report experiencing verbal violence in their work setting on a daily basis [14]. The data collection reported by Sefrin et al. does not specify whether sexual, racist or religious forms of harassment are included

Of the 86 participants reporting physical violence experience in the past year, the majority stated that these cases occurred during an assignment (*n* = 86), without dangerous objects or weapons being involved (*n* = 83) but with the involvement of alcohol and/or the influence of substances (*n* = 72) and with the patient being the main perpetrator (*n* = 78). Approximately a third of participants reported having their employer notified about all cases of violence (*n* = 28). Physical violence perpetrated by colleagues was reported by five participants.

Of the 129 participants who described experiencing non-physical violence in the past year, the majority also reported the cases to have taken place during an assignment (*n* = 86), with patients as perpetrators (*n* = 113), including the influence of alcohol and/or substances (*n* = 98) but also sometimes not involving any substances (*n* = 96). Only eight participants reported that they notified their employer in cases of non-physical violence. Notably, 40 participants reported having experienced non-physical violence perpetrated by colleagues and eight of those stated that 100% of their experiences of non-physical violence in the past 12 months stemmed from colleagues’ actions.

We examined which factors were significantly associated with participants having experienced physical and non-physical violence in the workplace in the past 12 months. As indicated by the Chi^2^ test result (Table 1), gender showed a statically significant association with the experience of physical violence, where women had a higher probability of reporting the experience of physical violence compared to men (OR = 2.214, 95%CI 1.209–4.054, Table 3).

In the case of non-physical violence, several sociodemographic variables and items demonstrated a significant association with the likelihood of having experienced non-physical violence in the past 12 months. Participants aged between 45 and 49 years were less likely to have experienced non-physical violence in the past 12 months (OR = 0.291, 95%CI 0.104–0.817) compared to participants younger than 24 years. Participants who had been working for their current employer for 16 years or more had a significantly lower likelihood of having experienced non-physical violence in the past 12 months (OR = 0.339, 95%CI 0.124–0.929) compared to participants who have been with their current employer for a year or less. Furthermore, respondents without children were more likely to have experienced non-physical violence in the past 12 months (OR = 2.623, 95%CI 1.463–4.704). Despite the significant associations observed here, it needs to be noted, that all the models’ fits were rather suboptimal with all Nagelkerke scores being below 0.100.

We explored the possibility of associations between the perceived tendency of violence towards emergency personnel and having had experiences of violence in the workplace in the past 12 months (Table 4). Participants who had experienced physical violence in the past 12 months had a higher likelihood of perceiving an increase in the prevalence of violence against emergency personnel (OR = 6.766, 95%CI 3.798–12.053). Considering non-physical violence, participants with experiences in the past 12 months had a higher likelihood of perceiving an increase in the prevalence of violence against emergency personnel (OR = 1.688, 95%CI 1.007–2.830).

**Table 3:** Factors associated with violence experiences in the past 12 months. Binomial logistics regression testing for both variables for violence experiences

	Have you experienced physical violence at work in the last 12 months?			Have you experienced non-physical violence at work in the last 12 months?		
	No	Yes	OR (95%), *p* value	No	Yes	OR (95%), *p* value
Age						
Younger than 24 years	31	20	Ref. *Nagelkerke* = *0.044*	18	33	Ref. Nagelkerke = 0.078
25–29 years	29	27	1.443(0.669–3.112) *p* = 0.350	17	39	1.251(0.557–2.810) *p* = 0.587
30–34 years	25	10	0.620(0.246–1.562) *p* = 0.311	15	20	0.727(0.301–1.757) *p* = 0.479
35–39 years	14	7	0.775(0.267–2.253) *p* = 0.640	8	13	0.886(0.310–2.537) *p* = 0.822
40–44 years	10	10	1.550(0.547–4.391) *p* = 0.409	11	9	0.446(0.156–1.277) *p* = 0.133
45–49 years	15	8	0.827(0.296–2.306) *p* = 0.716	15	8	0.291(0.104–0.817) *p* = 0.019
50 years or older	14	4	0.443(0.127–1.538) *p* = 0.200	11	7	0.347(0.115–1.051) *p* = 0.061
Gender						
Male	110	55	Ref. *Nagelkerke* = *0.040*	75	90	Ref. *Nagelkerke* = *0.014*
Female	28	31	2.214(1.209–4.054) *p* = 0.010	20	39	1.625(0.874–3.021) *p* = 0.125
Immigration background						
No	129	83	Ref. *Nagelkerke* = *0.012*	90	122	Ref.* Nagelkerke* = *0.033*
Yes—born in the EU	6	1	0.259(0.031–2.190) *p* = 0.215	1	6	4.426(0.524–37.412) *p* = 0.172
Yes—born in Germany with family migration background	3	2	1.036(0.170–6.333) *p* = 0.969	4	1	0.184(0.020–1.678) *p* = 0.133
Yes—born outside the EU	0	0		0	0	
Province°						
Bayern	115	75	Ref. *Nagelkerke* = *0.004*	82	108	Ref. *Nagelkerke* = *0.003*
Other	23	11	0.733 (0.338–1.592) *p* = 0.433	13	21	1.226(0.580–2.594) *p* = 0.593
Type of emergency service area						
Predominantly rural	21	12	Ref. *Nagelkerke* = *0.001*	14	19	Ref. Nagelkerke = 0.039
Predominantly urban	29	18	1.086(0.432–2.729) *p* = 0.860	13	34	1.927(0.752–4.937) *p* = 0.172
Rural and urban (mixed)	40	26	1.137(0.479–2.699) *p* = 0.770	34	32	0.693(0.299–1.610) *p* = 0.394
Exclusively urban (incl. metropolitan)	48	30	1.094(0.471–2.542) *p* = 0.835	34	44	0.954(0.419–2.171) *p* = 0.910
Qualification (binary)						
Non-paramedic			Ref. *Nagelkerke* = *0.003*	43	55	Ref. *Nagelkerke* = *0.001*
Paramedic			1.224(0.710- 2.111) *p* = 0.467	52	74	1.113(0.652–1.897) *p* = 0.695
Years of experience in emergency medicine*						
Less than 1 year	6	0	*Pearson Chi*^*2*^ = 0.390*	2	4	Ref. *Nagelkerke* = *0.059*
2–4 years	31	21		17	35	1.029(0.171–6.188) *p* = 0.975
5–9 years	40	28		25	43	0.860(0.147–5.036) *p* = 0.867
10–15 years	20	13		13	20	0.769(0.123–4.821) *p* = 0.779
More than 16 years	41	24		38	27	0.355(0.061–2.081) *p* = 0.251
Years of working for current employer						
Less than 1 year	18	9	Ref. *Nagelkerke* = *0.008*	10	17	Ref. *Nagelkerke* = *0.062*
2–4 years	37	28	1.514(0.592- 3.870) *p* = 0.387	21	44	1.232(0.482–3.150) *p* = 0.662
5–9 years	37	24	1.297(0.501–3.357) *p* = 0.592	26	35	0.792(0.312–2.010) *p* = 0.623
10–15 years	19	11	1.158(0.389–3.450) *p* = 0.792	12	18	0.882(0.303–2.571) *p* = 0.819
More than 16 years	27	14	1.037(0.371–2.899) *p* = 0.945	26	15	0.339(0.124–0.929) *p* = 0.035
Mode of employment						
Full-time	87	61	Ref. *Nagelkerke* = *0.011*	64	84	Ref. *Nagelkerke* = *0.001*
Part-time°°	45	23	0.729(0.400–1.328) *p* = 0.301	28	40	1.088(0.608–1.948) *p* = 0.775
Voluntary work	6	2	0.475(0.093–2.435) *p* = 0.372	3	5	1.270(0.293–5.511) *p* = 0.750
°°Part-time working hours in percentage (*n* = 68)						
< = 20%	15	5	Ref. *Nagelkerke* = *0.030*	11	9	Ref. *Nagelkerke* = *0.048*
21–49%	18	12	2.000(0.574–6.968) *p* = 0.276	10	20	2.444(0.764–7.820) *p* = 0.132
50–79%	11	5	1.364(0.316–5.892) *p* = 0.678	6	10	2.037(0.532–7.794) *p* = 0.299
> = 80%	1	1	3.000(0.157–57.365) *p* = 0.466	1	1	1.222(0.067–22.401) *p* = 0.892
Family constellation						
Children	46	22	Ref. *Nagelkerke* = *0.009*	40	28	Ref. *Nagelkerke* = *0.063*
No children	92	64	1.455(0.798–2.650) *p* = 0.221	55	101	2.623(1.463–4.704) *p* = 0.001

*The binomial logistic regression could not be performed due to a null value in the reference category

Participants reported on their assessment of the likelihood that they would experience violence in their current workplace context. The results are presented in Supplementary Table 3 .

**Table 4:** Odds ratios for the association between personal violence experiences and the perception of violence trends in emergency services

In your opinion, has the frequency of violence in the emergency services increased or decreased in recent years?							
Have you experienced physical violence at work in the last 12 months?					Total, *N*		OR, 95% CI
	No, rather decreased or stayed the same*	There is no clear trend	Yes, slightly increased	Yes, significantly increased		Adjusted for gender, emergency service area**	Unadjusted***
No	39	21	56	22	138	Ref	Ref
Yes	9	3	20	54	86	6.766 3.798–12.053 *p* < 0.001	6.934 3.921–12.264 *p* < 0.001

Have you experienced non-physical violence at work in the last 12 months?					Total, *N*	OR, 95% CI	
	No, rather decreased or stayed the same*	There is no clear trend	Yes, slightly increased	Yes, significantly increased		Adjusted for gender, emergency service area**	Unadjusted***
No	27	12	29	27	95	Ref	Ref
Yes	21	12	47	49	129	1.688 1.007–2.830 *p* = 0.047	1.770 1.073–2.919 *p* = 0.025
	48 21.4%	24 10.7%	76 33.9%	76 33.9%	224 100%		

*For the purpose of the analyses, the first two categories were combined due to comparably lower frequencies; the detailed frequencies are as follows: „No, rather decreased“ = 3 (1.3%). „N,. rather stayed the same“ = 45 (20.1%)

**Omnibus Test p < 0.001. AIC = 217.670. BIC = 248.375 ***Omnibus Test *p* < 0.001. AIC = 61.284. BIC = 78.342

### Reporting procedures and behavior

Approximately a third of participants reported to have established procedures for reporting violence at their workplace (32.6%). Of those, the majority had not yet submitted or have not had the need to submit a report yet. Still, 35.6% have abstained from submitting a report after having experienced violence at least once („Yes, once“ = 10, „Yes, on multiple occasions“ = 16). When asked to provide details on the reporting procedure for violent occurrences, the majority of participants indicated a form of report either to their direct manager and employer or to other structures within as well as outside their immediate organization (55.9%). Further details on the constellation of the reporting procedure, e.g. written or verbal form, timing, utilization of certain platforms or programs, were not disclosed.

Additionally, we asked participants if they were actively being encouraged to report violent occurrences at their workplace. The responses were split in half, with the participants who stated that they were actively encouraged were mostly empowered to do so by their manager or employer (89.4%), their colleagues (61.9%) as well as family and friends (13.3%).

### Perceptions of the origin of violence and effective preventive measures

We asked participants for their opinions regarding the main triggers of physical and non-physical violence perpetrated against EMS personnel (Table 5). The questions were phrased neutrally, without providing any specifications about the source or setting of the violence to avoid suggestive framing and consequent confounding of the results.

**Table 5:** Frequency of themes mentioned in the free-text responses to the questions on the most relevant factors facilitating violence as well as on the most effective and desired preventive measures against violence

“In your opinion, what are the (three) most important factors that contribute to violence in your working environment?”	Contributing factors to physical violence (*n* = 212)	Contributing factors to non-physical violence (*n* = 205)
Working conditions in emergency medicine	23	49
Alcohol and substance use	123	80
Physical and mental overload with the situation (patients)	42	28
Lack of de-escalation competencies in EMTs/violence-inducing behavior of EMTs	35	27
Disrespect, aggressive behavior (general) of patients/patients'family and/or friends/others (incl. sexism, racism and other forms of discrimination)	69	51
Misunderstanding, miscommunication, false expectations	41	37
Disagreement with/anxiety of EMTs measures	19	7
Somatic or mental illness (patients)	22	20
Systematic issues (lack or insufficiency of preventive measures, medical care, psychosocial support)	2	5

“In your opinion, what are the (three)…”	…most effective measures to reduce violence in your working environment? (*n* = 209)	…measures you would like to see most urgently? (*n* = 203)
Improving the working conditions incl. work time, (team) motivation, managers'competencies and qualifications	38	56
Mental health support at work incl. stress management, awareness raising	25	18
Training in and implementation of de-escalation/communication techniques, crisis management incl. removing oneself from the situation/assignment	112	50
Training in self-defense techniques. provision of defense equipment (incl. Body cams)	19	39
Awareness-raising and prevention among the population about the work of EMTs	42	25
Active (preventive) involvement of the police and other security forces in certain cases	28	9
Tightening the (legal) protection from and/or consequences of acts of violence/violence; consistent reporting of every case	40	47
Better/more specific inquiry by the 112 operators	7	4

In terms of physical violence (*N* = 212), participants’ feedback referred mostly to alcohol and substance use (*n* = 123), followed by general disrespect, aggressive behavior and discrimination (*n* = 69). Situational overload of the patients (*n* = 42) as well as miscommunication (*n* = 41) and lack of de-escalation competencies of EMS workers (*n* = 35) rounded up the five most frequent themes in the open-ended responses.

The most frequently mentioned issues regarding non-physical violence (*N* = 205), were also alcohol and substance use (*n* = 80), general disrespect, aggressive behavior and discrimination (*n* = 51), followed by the working conditions in emergency medicine (*n* = 49; Table 5). Miscommunication (*n* = 37) and the lack of de-escalation methods utilized by EMS workers (*n* = 27) concluded the five most frequent themes mentioned in the responses.

To prevent violence from occurring (*N* = 209), participants found the training in and implementation of de-escalation and communication techniques to be the most effective measures (*n* = 112; Table 6). However, the most urgent actions desired by participants (*N* = 203) are the improvement of the working conditions in emergency services incl. work time, (team) motivation, managers' competencies and qualifications (*n* = 56).

**Table 6:** Availability of established procedures for reporting violence in the workplace and their utilization reported by participants

	Yes			No
Reporting procedures				
Are there established procedures for reporting violence in your workplace?	73 (32.6%)			151 67.4%
If yes, what procedures are in place in your workplace? (*N* = 59*)				
Internal (Digital) reporting system	23 (39.0%)			
Reporting via the operational protocol (specifically for non-physical violence)	1 (1.7%)			
Reporting to the manager/association/union/Medical Director Rescue Service, other structures incl. police	33 (55.9%)			
Other	3 (5.1%)			
Unknown	3 (5.1%)			
Reporting behavior	Yes, once	Yes, on multiple occasions	No	
If yes: Have you already submitted a report via the respective procedures?	9 (4.0%)	16 (7.1%)	48 (21.4%)	
If yes: Should you have already submitted a report via the relevant procedures, but decided not to?	10 (4.5%)	16 (7.1%)	47 (21.0%)	
Are you being encouraged to report violence and violence at your workplace	Yes			No
	113 (50.4%)			105 (46.9%)
If yes, from whom:				
Manager/employer	101 (89.4%)			
Colleagues	70 (61.9%)			
Trade union	15 (13.3%)			
Association	1 (0.9%)			
Own family/friends	29 (25.7%)			
Others	6 (5.3%)			

Utilization was reported only by participants that have stated to have established procedures at their workplace

## Discussion

In our study, we present data on the frequency and parameters of violence experienced by EMS workers, primarily in Bavaria. This includes factors associated with an increased likelihood of reporting violence as well as the personal experience of violence enhancing the perception that violence is increasingly prevalent. In addition, we present qualitative data on the most relevant factors driving violence, according to the EMS workers’ vision as well as on the most efficient and urgently desired preventative measures.

In line with previous studies, non-physical violence occurred at a higher rate among our survey participants, where the perpetrators were primarily patients and the incidents occurred during assignments [4, 15–17]. Age was demonstrated to be a significant factor influencing the frequency of self-reported experiences of violence. Although we cannot determine with certainty the nature of the interaction between age and workplace violence, a decreased rate of violence experienced by personnel correlated to increasing age has been published before [18, 19]. We were not able to monitor similarly overlapping results in terms of gender, as previous studies have primarily observed a higher proportion of reported or experienced violent incidents, especially physical violence, associated with the male gender [18, 20, 21].

Possible explanations for this difference may be examined through a higher awareness of violence among female EMS members as well as, as previously observed in the context of psychiatric care, male employees potentially assuming violent behavior by patients and unconsciously exerting an attitude which assumes potential aggression [18, 20, 22]. Furthermore, our findings may correspond to the high rate of workplace violence experienced by female nurses, which is well documented [19, 23, 24]. Additional data collection and analyses are required to determine the exact mechanism of interaction between gender and workplace violence perpetrated against EMS personnel.

Participants also reported experiencing physical and more often non-physical violence by colleagues. Although the topic of violence between colleagues and staff is not well examined in the context of pre-hospital medicine, our findings mirror those of previous examinations in the context of medical residencies and internships, concluding that younger and less experienced staff members are particularly exposed to non-physical violence from their colleagues and supervisors [17, 18, 25–28].

Violence perpetrated by colleagues and supervisors is often left undocumented due to concerns about possible negative repercussions and embarrassment [17, 25, 28]. We were not able to explore associations between violence and racial or ethnic characteristics since the majority of participants indicated no background of immigration nor family history.

### Reporting procedures and behavior

We observed a lack of established procedures and platforms for reporting workplace violence in EMS. Underreporting of violent occurrences is one of the major impediments to systematically accessing data on the topic and can be observed in up to 80% of cases, with non-physical violence tending to be less frequently reported than physical [4, 6, 11, 19]. Berger et al. disclose that indeed the absence or insufficiency of reporting mechanisms significantly increases underreported violence [4]. This result places an emphasis on the discrepancy between the self-reported frequency of violence experienced by the survey’s participants and the frequency of case reports using the established procedures even when available. Unwillingness to report violent incidents has been observed when health care workers (HCWs) believe it to be insignificant either in terms of magnitude or potential consequences for the perpetrator or, as stated above, often when the hostile behavior originates from a colleague or supervisor [4, 17, 25, 28].

Previous analyses have noted a significantly lower likelihood of violent occurrences in departments and organizations with established reporting mechanisms, particularly in organizations with strict zero-tolerance policies [11, 19]. A binding commitment by the employer against all forms of violence, especially when enforced through an accessible reporting procedure, may encourage employees to report incidents. Implementing zero-tolerance policies against violence and abuse against HCWs is also embedded as a central strategy for retaining and recruiting HCWs in the WHO’s European Region 2023–2030 Framework for action on the health and care workforce [29].

Participants who had reported violent incidents in the workplace perceived a general increase in violence compared to those who hadn’t. This perception, however, may also be partially attributed to an increased awareness about workplace violence. Previous interventional studies have also documented a self-reported increase in violent incidents instead of a decrease following the implementation of preventive measures and reporting procedures [30, 31]. These studies thus indicate the significance of violence awareness and reporting procedures as critical factors in data collection as well as in the prevention of workplace violence in healthcare.

### Working conditions, including team members and leadership

The responses to our survey showcase the relevance of appropriate working conditions for EMS workers. In a previous nationwide survey, German EMS workers attributed their below-average job satisfaction primarily to organizational and managerial issues as well as to low payment [32]. Specifically, communication issues between managerial levels as well as between management and employees demonstrate a consistently high placement among EMS workers’ stressors and dissatisfaction at the workplace [32, 33].

The prioritization of working conditions as a factor for EMS workers matches with available evidence on appropriate conditions, camaraderie and support at the workplace being predictors of job satisfaction and lower turnover [34]. Specifically, support from the respective supervisor as well as by colleagues has shown to be significantly associated with job satisfaction [35–37]. Encouragement and empathy from leadership especially in the context of conflict may thus significantly improve EMS workers’ satisfaction and retention [6].

### Preventive measures and interventions

In terms of primary and secondary prevention of violence in the workplace, our results present an inclination towards communication-based approaches that prevent and manage violent situations. Indeed, interventions aimed at improving risk assessment, de-escalation and resilience have shown to improve healthcare workers’ self-efficacy and confidence in preventing and managing violent situations in the workplace [9, 34, 38]. These competencies are particularly relevant in the context of assignments involving alcohol and drug use, as the influence of substances has been identified by the participants as the primary cause of violence [5, 39, 40]. Additionally, and in consideration of the response category of disrespect and generally aggressive behavior, appropriate de-escalation techniques may significantly contribute to managing the situation in the safest manner for all participants.

In terms of the educational format of teaching de-escalation and conflict management techniques, simulation-based training has shown to significantly facilitate the competence to predict a violent incident as well as the confidence in handling one [41].

Against the background of previously published evidence, our results highlight the perceived effectiveness of communication-based approaches towards workplace violence mitigation in EMS as their adoption would be met by workers’ willingness to participate. However, the prominent placement of improving managerial competencies as a preventive measure against violence is particularly noteworthy. Matched against the perceived origins of violence by participants, the prominent positioning of leadership competencies’ development as a preventive measure highlights the already eminently underscored need of EMS workers for a conscious working environment defined by supportive colleagues and managers. This guides the discussion towards the exhaustive benefits of an employer’s binding commitment towards a violence-free workplace, ideally reinforced with established procedures and mechanisms, and draws a full circle back to the role of EMS organizations as employers actively, although probably to an extent unknowingly, affecting EMS workers’ perception of the workplace as well as their job satisfaction.

### Limitations

We are unable to report a response rate based on the targeted population for two main reasons. Firstly, we do not have access to reliable information on the number and constellation of EMS personnel in Germany. However, national-level data from 2021 indicates a lower representation of female EMS workers in our sample (34% vs. 25% in the survey’s sample) [42].

Secondly, the weak response led us to disseminate the call for participation via social media platforms (e.g. Facebook), which further impeded our assessment of the addressed population in terms of size, occupational and sociodemographic profile. A low response rate has previously been observed in other survey-based studies on violence in EMS [9].

Additionally, despite our attempt to phrase the title of the invitation to the study in the most neutral manner, a certain sampling bias cannot be excluded. A potential preferential participation of people with either a particularly good or a particularly bad perception of the working conditions in emergency medicine in Germany is possible. This limitation needs to be taken into account, especially against the background of working conditions being placed by participants as a top priority in the adoption of measures.

Finally, the cross-sectional retrospective design of our study replicates the concept of previously published examinations of the topic. However, a longitudinal prospective design with data collection methods intertwined with the established assignment protocols in EMS may provide more reliable data as memory-based biases will be better controlled for. Additionally, more qualitative data on the causes and context of workplace violence in EMS is urgently needed. In our study we identified a multitude of layers in the perpetrators’ backgrounds, motives, actions and the perception of EMS workers experiences of violent incidents in specific cases as well as on workplace violence in general. Essentially, our qualitative findings indicate structural and systematic areas of concern for the interpersonal, managerial and operational aspects of EMS organizations. These need to be examined thoroughly to provide an exhaustive overview of the organizational issues in EMS and define the matching measures to prevent all forms of violence and facilitate workers’ job satisfaction, motivation and retention.

However, despite certain limiting factors, our findings provide valuable insights into the frequency and perception of violence in EMS in Germany. The results documenting workplace violence perpetrated by colleagues as well as on reporting procedures and behavior highlight the need for organizations to adopt a strict position against all forms of violence, accompanied by accessible and transparent reporting mechanisms. Further detailed evidence is needed to enable the recommendation of effective strategies and practices in secondary prevention of violent occurrences, specifically considering tailored de-escalation and situation management capacity-building.

## Conclusion

Experiences of physical and non-physical violence are prevalent among EMS workers in Germany and enhance their perception of violence occurring in the context of their work. Considering the insufficient availability and implementation of mechanisms for reporting violence, employers need to commit towards employees’ mental and physical health beyond the legal obligation by establishing comprehensive strategies against all forms of violence in the workplace as well as clear mechanisms for reporting cases. As our results indicate, measures concerning de-escalation, communication, and improving working conditions would particularly appeal to EMS workers’ needs.

## Supplementary Information

Below is the link to the electronic supplementary material.

Supplementary file1 (PDF 756 kb)

## Data Availability

All data are available from the corresponding author upon reasonable request. An abstract related to the topic of this paper was presented at the 17 th European Public Health Conference by the first author of the hereby submitted manuscript: A Zhelyazkova, M Bonigut, Working in emergency services in Germany: cross-sectional data on violence experience and perception, *European Journal of Public Health*, Volume 34, Issue Supplement_3, November 2024, ckae144.1838, https://doi.org/10.1093/eurpub/ckae144.1838.
