# Supplementary material for: Violence in emergency services and preventative measures: results from an online survey from Germany
Source: Intern Emerg Med. 2025 Jun 6;21(2):693–704. doi: 10.1007/s11739-025-03994-4 (PMC13061800; doi:10.1007/s11739-025-03994-4)
Supplement: Supplementary file 1 — Supplementary file1 (PDF 756 kb) [file 11739_2025_3994_MOESM1_ESM.docx]

**Violence in emergency services and preventative measures: results from an online survey from Germany**

Ana Zhelyazkova^1*^, Matthias Bonigut^1^, Eva Jansen^2^

^1^ Institut für Notfallmedizin und Medizinmanagement (INM), LMU Klinikum, LMU München, Germany

^2^

Institut für Sozialmedizin, Epidemiologie und Gesundheitsökonomie der Charité — Universitätsmedizin Berlin

*Corresponding author

Ana Zhelyazkova, [Ana.Zhelyazkova@med.uni-muenchen.de](mailto:Ana.Zhelyazkova@med.uni-muenchen.de)

ORCID-IDs:

Ana Zhelyazkova: <https://orcid.org/0000-0002-8085-5003>

Matthias Bonigut: <https://orcid.org/0009-0000-9374-3337>

Eva Jansen: <https://orcid.org/0000-0001-9890-6641>

## **Supplementary Table 1: Parameters of the cases of physical violence experienced in the past 12 months. The results solely represent the feedback from participants that have previously replied with "Yes" to the filter question "Have you experienced physical violence in the workplace in the past 12 months?“. We asked participants „What percentage of the cases of physical violence you have experienced in the past 12 months had the following characteristics?“**

|  | Percent of experienced cases of **physical violence** with the respective characteristics* | | | | | | | | | | | N |
| --- | --- | --- | --- | --- | --- | --- | --- | --- | --- | --- | --- | --- |
| **Location** | **0** | **10** | **20** | **30** | **40** | **50** | **60** | **70** | **80** | **90** | **100** | **86** |
| At the rescue station /stand-by (not during assignment) | 12 | 1 |  |  |  |  |  | 1 |  |  |  | 14 |
| During assignment / in the field* |  |  |  | 3 |  | 1 |  |  | 2 | 1 | 79 | 86 |
| During a large-scale event | 6 | 5 | 5 | 5 | 1 | 2 | 1 |  |  |  | 2 | 27 |
| Assignment in a domestic setting | 5 | 6 | 6 | 6 | 3 | 25 | 1 | 4 | 2 | 1 | 5 | 64 |
| Assignment in a public setting |  | 1 | 3 | 4 | 1 | 27 | 8 | 4 | 4 | 2 | 25 | 79 |
| **Weaponry** | **0** | **10** | **20** | **30** | **40** | **50** | **60** | **70** | **80** | **90** | **100** | **86** |
| Case involving a dangerous object or weapon | 7 | 7 | 5 | 5 |  | 10 | 1 |  |  |  | 1 | 36 |
| Case not involving a dangerous object or weapon |  |  |  |  | 2 | 10 |  | 5 | 4 | 5 | 57 | 83 |
| **Violent actions involved in the case** | **0** | **10** | **20** | **30** | **40** | **50** | **60** | **70** | **80** | **90** | **100** | **86** |
| Bites, scratches and/or pinching | 4 | 3 | 6 | 5 | 2 | 8 | 4 | 4 | 3 | 1 | 7 | 47 |
| Knife stabs | 9 | 1 | 1 |  |  |  |  |  |  |  |  | 11 |
| Blows including slaps in the face | 5 | 6 | 4 | 5 | 3 | 16 | 2 | 2 |  | 2 | 17 | 62 |
| Shots | 13 |  |  |  |  |  |  |  |  |  |  | 13 |
| Sexual harassment | 5 | 2 | 3 | 2 | 4 | 9 | 1 | 1 | 1 | 2 | 1 | 31 |
| Sexual acts | 8 |  | 1 | 1 | 2 | 1 |  |  |  | 1 |  | 14 |
| Pushing and shoving | 4 | 2 | 2 | 7 | 8 | 16 | 3 | 4 | 3 | 2 | 14 | 65 |
| Kicking | 5 | 8 | 1 | 9 | 6 | 3 |  |  |  | 1 | 6 | 39 |
| Choking | 8 | 2 |  |  |  | 1 |  | 1 |  |  | 1 | 13 |
| **Perpetrator** | **0** | **10** | **20** | **30** | **40** | **50** | **60** | **70** | **80** | **90** | **100** | **86** |
| Patient |  | 1 | 3 | 2 | 1 | 17 | 3 | 9 | 5 | 4 | 33 | 78 |
| Patient’s family or friends | 5 | 9 | 6 | 4 | 1 | 12 | 1 |  |  |  | 5 | 43 |
| Other participants (e.g. driver involved in the incident) | 9 | 3 | 4 | 4 | 4 | 6 |  |  |  |  | 1 | 31 |
| Bypasser(s) | 7 | 5 | 3 | 3 | 2 | 2 | 2 |  |  |  | 3 | 27 |
| Colleague(s) (incl. manager) | 7 | 1 | 2 |  |  | 1 |  | 1 |  |  |  | 12 |
| **Perpetrator - characteristics** | **0** | **10** | **20** | **30** | **40** | **50** | **60** | **70** | **80** | **90** | **100** | **86** |
| Under alcohol and/or substance influence | 2 | 2 | 4 | 6 | 3 | 17 | 5 | 6 | 4 | 2 | 23 | 74 |
| Probably not under the influence of alcohol and/or substances | 2 | 4 | 6 | 8 | 4 | 15 | 1 | 3 | 2 | 1 | 10 | 56 |
| Neurologically conspicuous due to somatic or mental illness (e.g. Hypoglycaemia, seizure, stroke, psychosis | 3 | 6 | 4 | 7 | 2 | 3 | 1 | 4 | 3 |  | 4 | 37 |
| **Consequences** | **0** | **10** | **20** | **30** | **40** | **50** | **60** | **70** | **80** | **90** | **100** | **86** |
| Minor bodily injury | 5 | 9 | 2 | 1 |  | 11 | 2 | 1 | 2 |  | 21 | 54 |
| Serious bodily injury | 8 | 3 | 1 | 1 | 1 | 1 |  |  |  |  |  | 15 |
| Non-physical consequences incl. use of immediate assistance and psychosocial emergency care, e.g. crisis service | 14 | 1 | 1 | 1 |  | 2 |  | 1 | 1 | 1 | 2 | 23 |
| Non-physical consequences incl. use of psychological or psychiatric support, e.g. therapy, trauma outpatient clinic, etc. | 7 | 1 | 1 |  | 1 |  |  |  |  |  | 1 | 11 |
| Non-physical consequences without recourse to immediate assistance or psychological/psychiatric support | 13 | 4 | 3 | 2 | 1 | 4 | 1 | 2 |  |  | 12 | 41 |
| Incapacity for work (up to 3 days) | 10 | 3 | 1 | 1 |  | 2 |  |  |  |  | 2 | 19 |
| Incapacity to work (longer than 3 days) | 6 |  | 1 | 2 |  | 2 |  |  |  |  | 1 | 12 |
| No immediate consequences | 6 |  | 2 |  | 1 | 7 |  | 2 | 6 | 4 | 30 | 58 |
| **Reporting** | **0** | **10** | **20** | **30** | **40** | **50** | **60** | **70** | **80** | **90** | **100** | **86** |
| Has the manager or employer been notified? | 6 | 4 | 4 | 5 | 3 | 4 | 1 |  | 1 | 1 | 28 | 57 |
| Has the employer's liability insurance association been notified? | 5 |  | 2 | 3 |  | 3 |  |  |  |  | 7 | 20 |
| Have criminal charges been filed? | 7 | 3 | 4 | 2 | 2 | 5 |  |  |  | 2 | 11 | 36 |
| Have the perpetrators been convicted? | 9 | 1 | 3 | 1 |  | 1 |  |  |  |  | 2 | 17 |

**The answer options provided to participants were not mutually exclusive as participants were able to report data on cases experienced in different settings and under various circumstances. Answers were given on a slide bar in sections of ten percent. Values represent the number of people that have chosen the respective percentage, e.g. 9 participants have chosen „0%“ in the category „Have the perpetrators been convicted“.*

## **Supplementary Table 2: Parameters of the cases of non-physical violence experienced in the past 12 months. The results solely represent the feedback from participants that have previously replied with "Yes" to the filter question "Have you experienced non-physical violence in the workplace in the past 12 months?“. We asked participants „What percentage of the cases of non-physical violence you have experienced in the past 12 months had the following characteristics?“**

|  | Percent of experienced cases of non-physical violence with the respective characteristics* | | | | | | | | | | | N |
| --- | --- | --- | --- | --- | --- | --- | --- | --- | --- | --- | --- | --- |
| **Location** | **0** | **10** | **20** | **30** | **40** | **50** | **60** | **70** | **80** | **90** | **100** | **129** |
| At the rescue station /stand-by (not during assignment)* | 15 | 10 | 5 | 5 |  | 2 | 2 | 1 | 3 | 3 | 5 | 51 |
| During assignment / in the field* |  | 3 | 4 | 3 |  | 2 | 2 | 2 | 6 | 15 | 86 | 123 |
| During a large-scale event | 14 | 2 | 9 | 8 | 5 | 5 | 1 | 1 | 1 |  |  | 46 |
| Assignment in a domestic setting | 5 | 5 | 5 | 13 | 9 | 35 | 9 | 7 | 6 | 2 | 7 | 103 |
| Assignment in a public setting | 2 | 5 | 5 | 9 | 6 | 35 | 12 | 8 | 9 | 2 | 19 | 112 |
| **Violent actions involved in the case** | **0** | **10** | **20** | **30** | **40** | **50** | **60** | **70** | **80** | **90** | **100** | **129** |
| Abuse | 10 | 1 | 5 | 2 | 1 |  |  |  | 1 |  | 1 | 21 |
| Bullying | 6 | 12 | 6 | 4 | 3 | 3 | 1 | 2 | 1 |  | 4 | 42 |
| Harassment | 7 | 12 | 7 | 14 | 3 | 8 | 4 | 3 | 2 | 1 | 2 | 63 |
| Sexual harassment | 7 | 6 | 7 | 5 | 1 | 3 | 2 | 1 | 2 | 1 | 1 | 36 |
| Racist or religious harassment | 6 | 9 | 11 | 4 | 7 | 2 | 2 |  |  | 1 |  | 42 |
| Threats, insults |  | 7 | 7 | 6 | 6 | 13 | 7 | 12 | 13 | 7 | 44 | 122 |
| **Perpetrator** | **0** | **10** | **20** | **30** | **40** | **50** | **60** | **70** | **80** | **90** | **100** | **129** |
| Patient |  | 5 | 12 | 12 | 3 | 39 | 7 | 7 | 11 | 4 | 13 | 113 |
| Patient’s family or friends | 4 | 11 | 19 | 16 | 9 | 21 | 5 | 1 | 4 |  | 4 | 94 |
| Other participants (e.g. driver involved in the incident) | 9 | 16 | 14 | 6 | 1 | 6 | 1 | 1 | 1 |  | 2 | 57 |
| By-passer(s) | 6 | 12 | 10 | 9 | 6 | 6 | 3 | 2 | 1 |  | 3 | 58 |
| Colleague(s) | 5 | 14 | 2 | 3 | 3 | 4 | 2 | 1 | 2 | 1 | 8 | 45 |
| **Perpetrator - characteristics** | **0** | **10** | **20** | **30** | **40** | **50** | **60** | **70** | **80** | **90** | **100** | **129** |
| Under alcohol and/or substance influence | 2 | 5 | 8 | 14 | 6 | 27 | 9 | 6 | 7 | 5 | 11 | 100 |
| Probably not under the influence of alcohol and/or substances | 4 | 9 | 11 | 7 | 5 | 19 | 4 | 10 | 11 | 1 | 19 | 100 |
| Neurologically conspicuous due to somatic or mental illness (e.g. Hypoglycaemia, seizure, stroke, psychosis | 5 | 21 | 9 | 16 | 2 | 6 | 1 |  |  | 1 |  | 61 |
| **Consequences** | **0** | **10** | **20** | **30** | **40** | **50** | **60** | **70** | **80** | **90** | **100** | **129** |
| Non-physical consequences incl. use of immediate assistance and psychosocial emergency care, e.g. crisis service | 15 | 5 | 2 | 1 | 1 |  |  | 1 | 1 |  | 1 | 27 |
| Non-physical consequences incl. use of psychological or psychiatric support, e.g. therapy, trauma outpatient clinic, etc. | 18 | 3 | 2 | 1 | 1 |  |  |  | 1 |  |  | 26 |
| Non-physical consequences without recourse to immediate assistance or psychological/psychiatric support | 17 | 3 | 5 | 4 | 4 | 1 | 1 |  | 4 |  | 15 | 54 |
| Incapacity for work (up to 3 days) | 14 | 1 | 2 | 2 | 2 | 1 | 1 |  |  |  |  | 23 |
| Incapacity to work (longer than 3 days) | 13 | 2 |  |  | 2 | 1 |  |  |  |  | 2 | 20 |
| No immediate consequences | 5 | 1 | 2 | 4 |  | 2 | 2 | 6 | 6 | 5 | 73 | 106 |
| **Reporting** | **0** | **10** | **20** | **30** | **40** | **50** | **60** | **70** | **80** | **90** | **100** | **129** |
| Has the manager or employer been notified? | 14 | 9 | 8 | 4 | 2 | 6 | 2 |  |  |  | 8 | 53 |
| Has the employer's liability insurance association been notified? | 18 | 1 |  | 1 | 2 | 2 |  |  |  |  |  | 24 |
| Have criminal charges been filed? | 15 | 5 | 4 | 2 | 1 | 4 |  | 2 | 1 |  | 3 | 37 |
| Have the perpetrators been convicted? | 20 |  | 2 |  | 1 | 1 |  |  |  |  |  | 24 |

**The answer options provided to participants were not mutually exclusive as participants were able to report data on cases experienced in different settings and under various circumstances. Answers were given on a slide bar in sections of ten percent. Values represent the number of people that have chosen the respective percentage, e.g. 20 participants have chosen „0%“ in the category „Have the perpetrators been convicted“.*

## **Supplementary table 3: Participants’ assessment of the likelihood of them experiencing violence in their current workplace context.**

| Please give a personal assessment of the following questions. | Not at all | Rather not | partly | Somewhat | Very much |
| --- | --- | --- | --- | --- | --- |
| How likely are you to experience violence in your current workplace? | 31  13.8% | 56  25.0% | 59  26.3% | 46  20.5% | 32  14.3% |
| How likely is it that you experience physical violence at your current workplace? | 40  17.9% | 81  36.2% | 46  20.5% | 45  20.1% | 12  5.4% |
| How likely is it that you experience non-physical violence at your current workplace? | 18  8.0% | 46  20.5% | 66  29.5% | 55  24.6% | 39  17.4% |
| How likely is it that you experience sexual harassment at your current workplace? | 90  40.2% | 75  33.5 | 26  11.6% | 20  8.9% | 13  5.8% |
| How likely is it that you experience sexualized acts at your current workplace? | 99  44.2% | 74  33.0% | 30  13.4% | 17  7.6% | 4  1.8% |
| How likely is it that you experience violence from patients in your current workplace? | 4  1.8% | 45  20.1% | 72  32.1% | 69  30.8% | 34  15.2% |
| How likely is it that you experience violence from patients' family and/or friends at your current workplace? | 4  1.8% | 58  25.9% | 83  37.1% | 59  26.3% | 20  8.9% |
| How likely is it that you experience violence from colleagues, including managers, in your current workplace? | 93  41.5% | 84  37.5% | 24  10.7% | 16  7.1% | 7  3.1% |
| How concerned are you about violence from other people in your current work environment? | 50  22.3% | 68  30.4% | 46  20.5% | 46  20.5% | 14  6.3% |
